# Personal care product use patterns in association with phthalate and replacement biomarkers across pregnancy

**DOI:** 10.1038/s41370-023-00627-w

**Published:** 2024-01-04

**Authors:** Emma M. Rosen, Danielle R. Stevens, Amanda M. Ramos, Erin E. McNell, Mollie E. Wood, Stephanie M. Engel, Alexander P. Keil, Antonia M. Calafat, Julianne Cook Botelho, Elena Sinkovskaya, Ann Przybylska, George Saade, Alfred Abuhamad, Kelly K. Ferguson

**Affiliations:** 1 Epidemiology Branch, National Institute of Environmental Health Sciences, Durham, NC USA; 2 Department of Epidemiology, University of North Carolina-Chapel Hill, Chapel Hill, NC USA; 3 Division of Cancer Epidemiology and Genetics, National Cancer Institute, Bethesda, MD USA; 4 Division of Laboratory Sciences, National Center for Environmental Health, Centers for Disease Control and Prevention, Atlanta, GA USA; 5 Department of Obstetrics and Gynecology, Division of Maternal-Fetal Medicine, Eastern Virginia Medical School, Norfolk, VA USA; 6 Department of Obstetrics and Gynecology, University of Texas Medical Branch, Galveston, TX USA

**Keywords:** Phthalates, Epidemiology, Chemicals in products

## Abstract

**Background:**

Humans are exposed to phthalates, a class of non-persistent chemicals, through multiple products, including personal care and cosmetics. Associations between specific phthalates and product use have been inconsistent. However, determining these connections could provide avenues for exposure reduction.

**Objective:**

Examine the association between patterns of personal care product use and associations with phthalate and replacement biomarkers.

**Methods:**

In the Human Placenta and Phthalates Study, 303 women were enrolled in early pregnancy and followed for up to 8 visits across gestation. At each visit, women completed a questionnaire about product use in the prior 24 hours and contributed urine samples, subsequently analyzed for 18 phthalate and replacement metabolites. At early, mid-, and late pregnancy, questionnaire responses were condensed and repeated metabolite concentrations were averaged. Latent class analysis (LCA) was used to determine groups of women with similar use patterns, and weighted associations between group membership and biomarker concentrations were assessed.

**Results:**

LCA sorted women into groups which largely corresponded to: (1) low fragranced product use (16-23% of women); (2) fragranced product and low body wash use (22–26%); 3) fragranced product and low bar soap use (26–51%); and (4) low product use (7–34%). Monoethyl phthalate (MEP) urinary concentrations were 7–10% lower and concentrations of summed di(2-ethylhexyl) terephthalate metabolites were 15–21% lower among women in the “low fragranced product use” group compared to the population mean. Few other consistent associations between group and biomarker concentrations were noted.

**Impact statement:**

Personal care products and cosmetics are a known exposure source for phthalates and potentially represent one of the most accessible intervention targets for exposure reduction. However, in this analysis accounting for concurrent use and fragranced status of products, we did not find any use patterns that corresponded to universally lower levels.

## Introduction

Phthalates are non-persistent chemicals that are frequently used as plasticizers and solvents. Exposure to phthalates occurs through the consumption of food and beverages that have previously been in contact with phthalate-containing packaging and processing materials, exposure to products such as adhesives and sealants, and through the use of cosmetics and personal care products [1–3]. Specifically, certain phthalates including diethyl phthalate (DEP), are common in fragranced products where they function as scent stabilizers [4]. Because of gender differences in the use of personal care and fragranced products, women frequently have higher concentrations of phthalate exposure biomarkers than men [5]. Women of reproductive age are frequent users of personal care products and cosmetics, and exposure is particularly concerning during pregnancy, as phthalate exposure has been associated with adverse outcomes including preterm birth and fetal growth restriction [6, 7].

Compared to other exposure sources, such as household applications (e.g., wallpapers, vinyl flooring, etc.), diet, and medical products and devices, cosmetic and other personal care product use may be more easily modifiable. While diet is an important source of phthalate exposure [8], it may be harder to modify compared to personal care products as exact sources of exposure are unclear and prior interventional studies have yielded mixed results [9]. Prior studies have examined associations between self-reported product use and phthalate exposure biomarkers, both in non-pregnant and pregnant populations [10–18]. These populations have varied with regard to socioeconomic status, geographic location, method of query, and types of products included. Positive associations between certain personal care and cosmetic products with specific phthalate biomarkers have been noted, but results are largely inconsistent across studies [19]. This may be a result of the differences in study populations described above, as well as temporal changes in product formation, differences in advertising of products, and local availability of products. Additionally, these differences may be attributable to the fact that most studies examine personal care products one at a time without consideration of simultaneous product use, though they are frequently used together. Patterns of product use may more accurately represent behaviors and account for confounding by other products; furthermore, changing behaviors to avoid patterns of product use could be more effective than avoiding single products.

We sought to examine patterns of personal care product use and associations with phthalate and replacement exposure biomarkers in the Human Placenta and Phthalates Study (HPP), a diverse cohort of pregnant women with repeated measures of exposure biomarkers and questionnaire information on personal care product use from 8-time points in gestation. The objectives of this study were to: (1) identify groups of individuals with similar patterns of personal care product use in early, mid-, and late pregnancy; (2) examine how product use patterns change across gestation; and (3) relate pregnancy period-specific group membership to urinary concentrations of phthalate and replacement biomarkers.

## Methods

### Study population

Women were enrolled in the Human Placenta and Phthalates Study prior to 14 weeks’ gestation from prenatal clinics at Eastern Virginia Medical School (EVMS) and the University of Texas Medical Branch (UTMB) from 2017–2018. Women were eligible if they were between the ages of 18–50, carrying a singleton pregnancy, and did not have any detected abnormalities in the fetus, umbilical cord, or in placental location within the uterus. Additional detail on this study has been previously described [20]. Between enrollment and 16 weeks’ gestation, women attended visits every 2 weeks (median 13, 15, 17 weeks). From 16 weeks’ gestation through delivery, women attended clinic visits every 4 weeks (median 21, 25, 29, 33, 37 weeks). At these 8 study visits, 303 women contributed at least one urine sample and completed a product use questionnaire. On average, women contributed 6.5 samples. Procedures were approved by the Institutional Review Boards at EVMS and UTMB and all participants signed informed consent forms prior to participating. Analysis of de-identified samples by the Centers for Disease Control and Prevention (CDC) laboratory was determined not to constitute engagement in human subjects research.

### Personal care product questionnaires

At each visit, women completed a questionnaire that asked them to select products from a list that they had used in the prior 24 hours. The list included vitamins and supplements, personal care products, cosmetics, insect repellants, air sprays, and cleaners. This analysis is restricted to personal care products and cosmetics, which have been linked to phthalate exposure biomarkers most consistently in the literature [19]. The full list of personal care products queried is included in Supplemental Table 1. Where relevant, women also reported if the product used was fragranced, fragrance-free, or the fragrance status was unknown. The questionnaire did not ask about specific brands or formulations.

Due to variability in sample size across study visits, we condensed responses from the 8 visits down to 3, corresponding to early, mid-, and late pregnancy, hereafter referred to as pregnancy periods. Visits 1 and 2 (gestational weeks 13–15) were considered early pregnancy, visits 3–5 (gestational weeks 17–25) were mid-pregnancy, and visits 6–8 (gestational weeks 29–37) were grouped as late pregnancy. Product use responses for the pregnancy periods were populated using the first completed response from the relevant visits (Supplemental Fig. 1). Women were only dropped from the LCA if they had completely blank questionnaire responses and there were no notable demographic differences between women who completed a questionnaire and those who did not.

### Quantification of phthalate and replacement metabolites

Urine samples were collected from each woman in sterile polypropylene specimen cups at each visit. Women were instructed not to use any wipes prior to sampling. Specific gravity (SG) was measured using a PAL -10S refractometer. Samples were stored at –80 °C until being shipped overnight and on dry ice to the National Center for Environmental Health laboratory at CDC for analysis. At CDC, urine was stored at or below –40 °C until analysis. Additional detail on collection processes has been previously described [20]. Quantification of phthalate and replacement metabolites involved enzymatic hydrolysis of the metabolites from their conjugated form, automated online solid phase extraction, separation with high-performance liquid chromatography, and detection using isotope-dilution tandem mass spectrometry [21]. The following metabolites were measured: monoethyl phthalate (MEP), mono-n-butyl phthalate (MBP), mono-hydroxybutyl phthalate (MHBP), mono-isobutyl phthalate (MiBP), mono-hydroxy-isobutyl phthalate (MHiBP), monobenzyl phthalate (MBzP), mono-3-carboxypropyl phthalate (MCPP), mono-2-ethylhexyl phthalate (MEHP), mono-2-ethyl-5-hydroxyhexyl phthalate (MEHHP), mono-2-ethyl-5-oxohexyl phthalate (MEOHP), mono-2-ethyl-5-carboxypentyl phthalate (MECPP), mono oxononyl phthalate (MONP), mono carboxyisooctyl phthalate (MCOP), and mono carboxyisononyl phthalate (MCNP). Additionally, four metabolites of replacements were measured: mono-2-ethyl-5-hydrohexyl terephthalate (MEHHTP), mono-2-ethyl-5-carboxypentyl terephthalate (MECPTP), both metabolites of di(2-ethylhexyl) terephthalate (DEHTP), and cyclohexane-1,2-dicarboxylic acid, monohydroxy isononyl ester (MHiNCH), and cyclohexane-1,2-dicarboxylic acid, monocarboxy isooctyl ester (MCOCH). Instrument-reading concentrations below the limit of detection (LOD) were retained if they were >0 and concentrations reported as 0 (i.e., absence of analytical signal) were imputed using LOD/√2, a method shown to be valid in situations with levels of values < LOD similar to what was present in our study [22]. We did not impute instrument-read values < LOD [23, 24].

We calculated molar sums for metabolites of the same parent compounds by summing the molar concentrations (nmol/mL) [20, 25]: MEHP, MEHHP, MEOHP, and MECPP for the sum of di(2-ethylhexyl) phthalate metabolites (∑DEHP); MCNP and MCOP for the sum of di-isononyl phthalate metabolites (∑DiNP); MBP and MHBP for the sum of di-n-butyl phthalate metabolites (∑DnBP); MiBP and MhiBP for the sum of di-iso-butyl phthalate metabolites (∑DiBP); MHiNCH and MCOCH for the sum of 1,2-cyclohexane dicarboxylic acid, diisononyl ester metabolites (∑DiNCH); and MEHHTP and MECPTP for the sum of DEHTP metabolites (∑DEHTP). Molar concentrations were then converted to ng/mL by multiplying the molar sum by the molecular weights of MECPP, MCOP, MBP, MiBP, MHiNCH, and MECPTP, respectively.

All phthalate and replacement exposure biomarkers were corrected for urine dilution using covariate-adjusted standardization in a model that included maternal age (continuous), gestational age at sample collection (continuous), pre-pregnancy body mass index (continuous), maternal education (3 level categorical), and maternal race and ethnicity (3 level categorical) [26, 27]. Natural log-transformed SG was modeled as a function of the above covariates to generate predicted SG values. Exposure biomarkers were then divided by the ratio of observed SG to predicted SG. The urine dilution standardization was conducted after the calculation of the molar sums. Maternal age, education, height, and race and ethnicity were self-reported at enrollment. Maternal weight was extracted from medical records and gestational age at sample collection was recorded as part of the study protocol.

To create more stable estimates of exposure within pregnancy periods, we condensed phthalate and replacement biomarker concentrations from the 8 visits into 3 time points, using the same timing scheme described for the questionnaire. We calculated the geometric mean of all available measurements within each pregnancy period, and, if only one concentration was available, that was used in place of the geometric mean.

### Covariates

Maternal race was self-reported with options of Caucasian, Black or African-American, Asian, Native Hawaiian/Other Pacific Islander, or “other.” Women also indicated their ethnicity as Non-Hispanic/Latina, Hispanic/Latina, or “refuse to disclose.” We created a composite race/ethnicity variable with levels of “non-Hispanic White”, “non-Hispanic Black”, “Hispanic, any race”, and “Asian, Native Hawaiian/Other Pacific Islander, or Other.” Highest education achieved was captured as high school graduate or below; some college, technical school, or associates degree; 4-year college degree. Insurance status was categorized using enrollment values as private, or self-pay/uninsured/government-assisted.

### Statistical analysis

All analyses were conducted in SAS 9.4 (Cary, NC, USA). We examined distributions of phthalate and replacement biomarker concentrations by pregnancy period, using SG-corrected and period-averaged values.

Latent class analysis (LCA) was used to identify groups of individuals with similar patterns of personal care product use among participants within each pregnancy period using PROC LCA in SAS 9.4. The number of classes was selected based on model fit statistics like Akaike and Bayesian Information Criterion, membership percentages, posterior probabilities, and interpretability of classes [28]. To reduce the number of items included in the LCA model, we used an a priori cut-off to drop items with less than <10 women reporting use at each visit (Supplemental Table 1). The remaining products were evaluated based on whether they had previously been associated with phthalates in the literature. If a product was lowly used, not previously linked to phthalates, or strongly racially colinear, it was excluded. Additionally, similar products were combined (e.g., conditioner and leave-in conditioner). In the cases of these composite variables, the participant was classified as a fragranced user if one instance of fragranced use was noted. Conversely, both products in a combined variable had to be reported as no use or fragrance-free use for a participant to be coded as those respective responses. For early pregnancy, responses were only available at one visit for 19-30% of women, varying by product. Of the remaining women, approximately 60% had the same responses for product use at each visit. In mid- and late pregnancy, approximately 40% of women had concordant values at all 3 visits, while an additional 30-40% had either a response only at one visit, or a response at two visits with those responses in agreement (data not shown).

Product responses were coded using one 3-level variable with levels corresponding to: no use, fragranced use (reported yes to use in the last 24 hours, and reported that the product was fragranced), and fragrance-free use (reported yes to use in the last 24 hours and reported that the product was fragrance-free). If the use of a product was reported but the participant also reported “fragrance unknown,” the response for the item was set to missing. If one product in a composite variable was indicated as “fragrance unknown”, we used the available response to determine the fragrance status. This was only applicable for the hand soap variable, as the other composite variables either did not have a fragrance component (i.e., cosmetics) or were not included in the main analysis (i.e., conditioner). Cosmetics and perfume were coded dichotomously as any use vs. none.

Using bar charts, we reported the number of identified groups in each pregnancy period, the proportion of total participants in each group, and the proportion of participants within each group who reported no use, fragrance-free use, or fragranced use of each personal care product included in the model. “Any use” for cosmetics and perfume was coded as “fragranced use” for the visualization only since the questionnaire did not specify whether these products were fragranced and responses had to be represented visually. Furthermore, perfume is, by nature, fragranced and many cosmetics include fragrance either to include a scent or to mask the scent of other ingredients [29].

Latent transition analysis (LTA) was then conducted to assess changes in class membership across pregnancy using PROC LTA. LTA uses data from all time points to determine class profiles and identify item response probabilities. Output of LTA includes conditional item response probabilities, prevalence of latent classes at each time point, and transition probabilities, representing the likelihood of membership in each given class conditional on membership in a specific class at the previous time point. We compared the demographic distributions of women who remained in the same product use groups between consecutive pregnancy periods and those who moved between groups.

To assess the relationship between product use group membership and measured phthalate and replacement biomarker concentrations, we compared mean biomarker concentrations across groups. Mean concentrations were calculated from linear regression generalized estimating equations. Models were weighted using inverse probability of treatment weights so that each latent class group was standardized to the distribution of covariates in the overall population at each pregnancy period. Thus, this weighting allowed a more direct comparison of mean urinary biomarker concentrations within groups to the overall population. Without weights, differences could be attributable to demographic differences across groups. Inverse probability weights were based on maternal race and ethnicity, age, highest educational achievement, and health insurance status. Women missing information on any of the included covariates were dropped from the model (*n* = 8, 7, 6 women at each point in pregnancy, respectively). Because product use variables representing the pregnancy period were derived from different visits for different items, it was not possible to align concentrations and product responses to be used from the same visit. As such, we used biomarker concentrations representing the entire pregnancy period.

To visualize the associations between product use group and biomarker concentrations, we created heat maps displaying the relative difference in biomarker concentration for women in the product use group compared to the period-mean concentration. Tables are separated by biomarkers of low molecular weight (LMW) phthalates, consisting of MEP, ∑DnBP, and ∑DiBP, high molecular weight (HMW) phthalate biomarkers, consisting of MBzP, MCPP, ∑DEHP, ∑DiNP, and MCNP, and replacement biomarkers, consisting of ∑DiNCH and ∑DEHTP.

### Sensitivity analysis

As a sensitivity analyses, we repeated analyses with the next best candidate models from the latent class analyses. Findings from LCA rely heavily on the user-selected model and rerunning analyses using an alternative model allows us to assess how much, if at all, conclusions change. We reran LCA using different subsets of products from the questionnaire to identify robustness of groups, as well as rerunning results retaining the “fragrance unknown” response instead of setting it to missing.

## Results

Of the 303 women recruited into this study, 258 had at least one completed questionnaire and measured biomarkers in early pregnancy (visits 1, 2), 268 in mid-pregnancy (visits 3–5), and 273 in late pregnancy (visits 6–8). Two hundred twenty-eight women had complete exposure and outcome information at all three-time points.

Just over half of the population was under 27 years old and approximately 39% of the women self-identified as non-Hispanic White, while 43% reported their race and ethnicity as non-Hispanic Black, and 16% as Hispanic (Table 1). Fifty-six per cent of women were married or living with their partner, 48% of women reported being unemployed at the time of enrollment, and a third of women reported smoking in the 3 months before pregnancy. Approximately 45% of women had a high school education or lower.

**Table 1:** Demographic characteristics of participants in the Human Placenta and Phthalates Study (*N* = 303).

	*N* (%)
**Race/ethnicity**	
Non-Hispanic White	118 (38.9)
Non-Hispanic Black	131 (43.2)
Hispanic, any race	49 (16.2)
Other^1^	5 (1.7)
**Clinic Site**	
EVMS	218 (72.0)
UTMB	85 (28.1)
**Marital status**	
Single^**2**^	131 (43.2)
Married or living with partner	166 (54.8)
* Missing*	6
**Current employment**	
None	139 (48.1)
Any	150 (51.9)
* Missing*	14
**Health insurance**^**3**^	
Private	82 (27.5)
Government-assisted	216 (72.5)
* Missing*	5
**Parity**	
0	108 (35.8)
1, 2	155 (51.3)
3+	39 (12.9)
**Smoking in 3 months prior to pregnancy**	
No	204 (67.8)
Yes	97 (32.2)
* Missing*	2
**Education**	
High school graduate or below	131 (44.7)
Some college, technical school, or associates degree	123 (42.0)
4-year college degree	39 (13.3)
* Missing*	10
**Age** (years)	
18–22	78 (25.9)
23–26.5	83 (27.6)
27–30	72 (23.9)
30–46	68 (22.6)
* Missing*	2
**Early pregnancy BMI** (kg/m^2^)	
<18.5	16 (5.3)
18.5–24.99	135 (44.7)
25–29.99	112 (37.1)
>30	39 (12.9)
* Missing*	1

*BMI* Body mass index; *EVMS* Eastern Virginia Medical School; *UTMB* University of Texas Medical Branch

^1^Includes Asian, Hawaiian/Pacific Islander, multiracial.

^2^Includes never married, divorced, separated, widowed.

^3^One participant who reported “self-pay/uninsured” was grouped with government-assisted.

Distributions of biomarker concentrations in this population have been previously defined [20]. Detection across all visits was >90% for all metabolites except MHBP, MCPP, MEHP, MHiNCH, and MCOCH (Supplemental Table 2-3).

Phthalate and replacement biomarker concentrations were relatively stable over pregnancy, with the exception of MEP and ∑DEHTP, both of which were highest in early pregnancy (Table 2).

**Table 2:** Median (IQR) of phthalate and replacement biomarkers^1^ (ng/mL) across pregnancy periods among women with information on personal care product use.

	Early pregnancy (*n* = 258)	Mid-pregnancy (*n* = 268)	Late pregnancy (*n* = 273)
**Phthalate biomarkers**			
MEP	58.5 (20.9, 128.6)	42.5 (17.3, 106.6)	44.5 (17.6, 100.8)
∑DnBP	12.0 (5.1, 24.8)	11.6 (4.6, 21.4)	11.5 (6.3, 23.9)
∑DiBP	11.2 (5.2, 20.9)	11.2 (6.5, 18.5)	12.0 (7.1, 23.2)
MBzP	3.2 (1.7, 7.3)	3.4 (1.7, 7.0)	3.6 (1.8, 7.4)
MCPP	1.1 (0.6, 1.7)	0.7 (0.4, 1.2)	0.7 (0.4, 1.2)
∑DEHP	15.5 (8.8, 28.4)	14.6 (9.6, 24.2)	15.4 (9.6, 23.0)
∑DiNP	5.5 (3.3, 9.8)	5.5 (3.3, 9.7)	5.5 (3.6, 9.1)
MCNP	1.2 (0.7, 2.1)	1.1 (0.7, 1.7)	1.0 (0.7, 1.8)
**Phthalate replacement biomarkers**			
∑DiNCH	1.4 (0.8, 2.7)	1.5 (0.9, 2.6)	1.2 (0.9, 2.3)
∑DEHTP	63.9 (25.4, 136.2)	46.2 (21.7, 89.2)	44.5 (22.7, 89.4)

*IQR* Interquartile range; *MEP* monoethyl phthalate; *∑DnBP* sum of di-n-butyl phthalate metabolites; *∑DiBP* sum of di-iso-butyl phthalate metabolites; *MBzP* monobenzyl phthalate; *MCPP* mono-3-carboxypropyl phthalate; *∑DEHP* sum of di(2-ethylhexyl) phthalate metabolites; *∑DiNP* sum of di-isononyl phthalate metabolites; *MCNP* mono carboxyisononyl phthalate; *∑DiNCH* sum of 1,2-cyclohexane dicarboxylic acid, diisononyl ester metabolites; *∑DEHTP* sum of di(2-ethylhexyl) terephthalate metabolites.

^1^Biomarker concentrations displayed are corrected for specific gravity and averaged within trimesters.

**Fig. 1:**
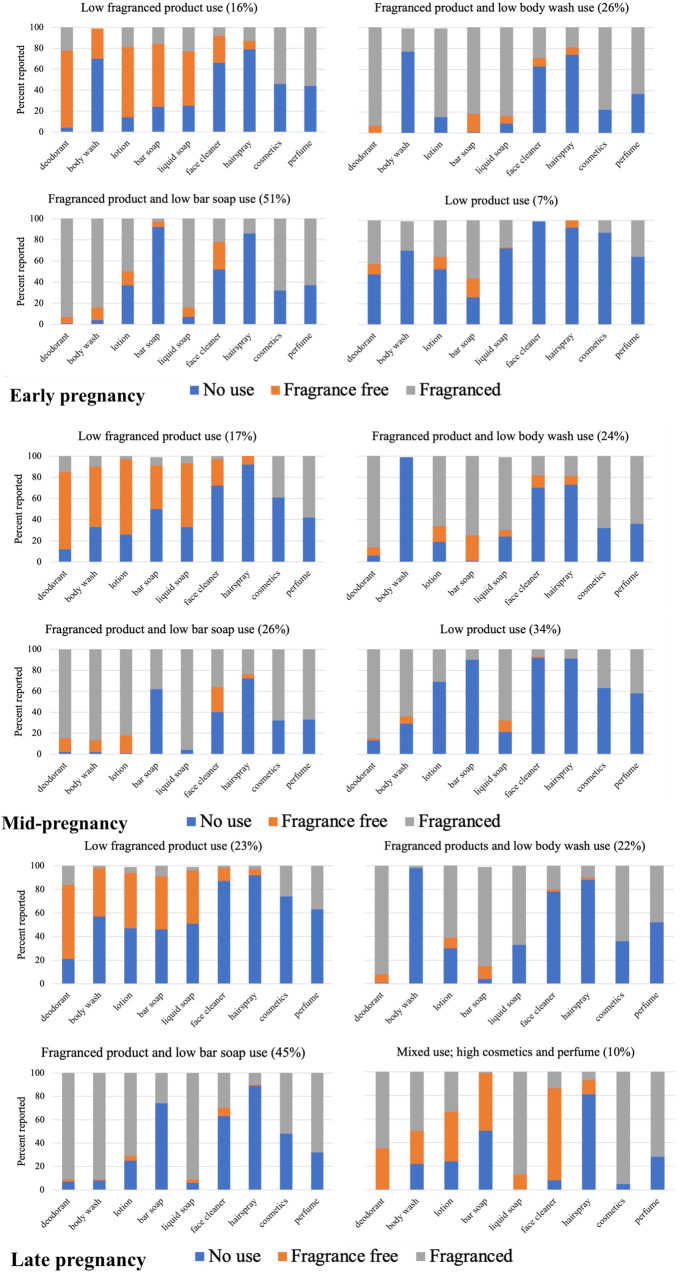
Use of individual personal care products within latent classes of participants in the Human Placenta and Phthalates Study. **A**–**C** represent early, mid-, and late pregnancy bar charts, respectively. Bar charts represent the four identified latent class groups at each period in pregnancy. Colors correspond to the proportion of women in each latent class who reported a certain type of product use.

LCA groups were constructed using questionnaire responses on use of the following products: deodorant, body wash, lotion, bar soap, liquid soap, face cleaner, hairspray, cosmetics, and perfume. We selected the 4-class model as the best fitting model across indicators and based on clarity of groups (Fig. 1). Fit statistics for various candidate models are displayed in Supplemental table 4. Three of the groups were replicated across all points in pregnancy, while one was characterized slightly differently in late pregnancy. The groups that were consistent across pregnancy were characterized and subsequently labeled using the most prominent trends in the groups to define them. These names corresponded to “low fragranced product use;” “fragranced product and low bar soap use;” and “fragranced product and low body wash use.” In the early and mid-pregnancy, the additional group was labeled as “low product use” while in late pregnancy, the profile was slightly different, and it was thus labeled as “mixed use with high cosmetics and perfume.”

The proportion of women in each group across pregnancy was more stable for some groups than others. Across pregnancy, the proportion of women in the “low fragranced product use” and “fragranced product and low body wash use” was relatively consistent (range: 16-23% and 22–26%, respectively). However, in the “fragranced product and low bar soap use” group, many fewer women were classified into that group in mid-pregnancy compared to early and late pregnancy. Conversely, a much higher percentage of women were in the “low product use” group at mid-pregnancy compared to early pregnancy (34 vs. 7%).

The results from the LTA analysis showed that most women remained in the same groups between early and mid-pregnancy (62–92% remaining in the same group) but less so between mid- and late pregnancy (Supplemental tables 5,6; supplemental Fig. 2). The latter was likely a function of having different patterns of personal care product use derived from the LCA in late pregnancy. Overall, the most stable group was “fragranced product and low bar soap use,” where 77% of women who were in that group at early pregnancy remained in that group in mid-pregnancy, and 84% of the women in that group in mid-pregnancy were also in that group in late pregnancy. Transition probabilities between early and late pregnancy were similar to those between mid- and late pregnancy (Supplemental table 7). Compared to the overall population, there were no meaningful demographic differences among women who transitioned between different product use groups in consecutive pregnancy periods (Supplemental table 8).

**Fig. 2:**
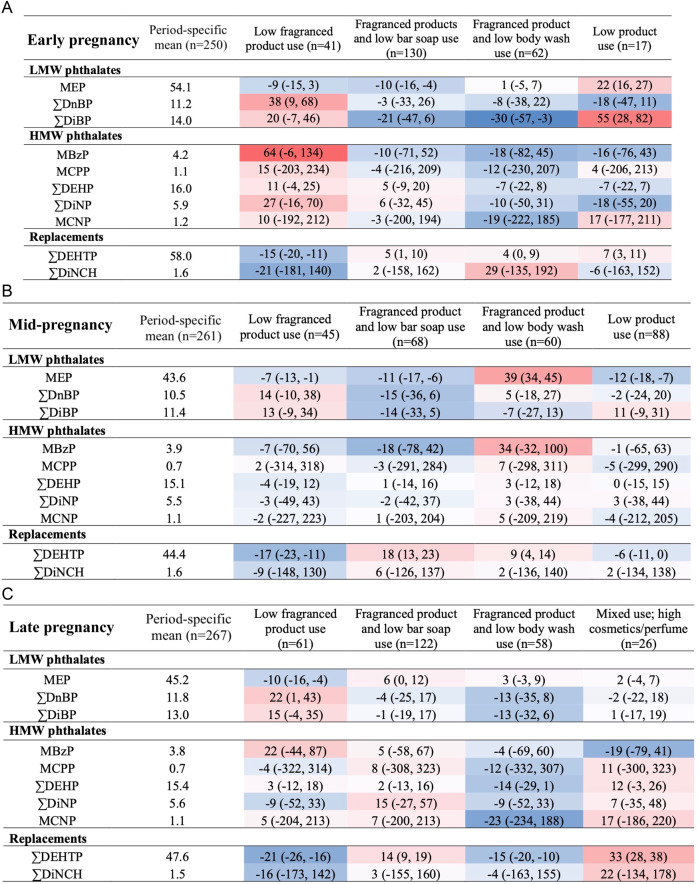
Percent difference in weighted^1^ mean phthalate and phthalate replacement biomarker concentrations2 (ng/mL) by latent class of personal care product use compared with weighted period-specific mean. **A**–**C** represent early, mid-, and late pregnancy tables, respectively. 1. Models were weighted for maternal age, highest education achieved, health insurance, self-reported race/ethnicity. 2. Biomarkers were transformed, standardized for specific gravity, and averaged within pregnancy period. *Indicates percent difference that is statistically significantly different from 0. Blue cells reflect a lower mean biomarker concentration among participants in the latent class group relative to the period-specific overall mean. Red cells reflect a higher mean biomarker concentration among participants in the latent class group relative to the period-specific mean. LMW low molecular weight, HMW high molecular weight, MEP monoethyl phthalate, ∑DnBP sum of di-n-butyl phthalate metabolites, ∑DiBP sum of di-iso-butyl phthalate metabolites, MBzP Monobenzyl phthalate, MCPP mono-3-carboxypropyl phthalate, ∑DEHP sum of di(2- ethylhexyl) phthalate metabolites, ∑DiNP sum of di-isononyl phthalate metabolites; MCNP mono carboxyisononyl phthalate, ∑DiNCH sum of 1,2-cyclohexane dicarboxylic acid, diisononyl ester metabolites, ∑DEHTP sum of di(2-ethylhexyl) terephthalate metabolites.

There was no product use group that had consistently higher or lower concentrations of all phthalate and replacement biomarkers over time (Fig. 2). However, we noted some patterns within LMW, HMW, and replacement biomarker groups. Estimates of percent difference were frequently imprecise, especially for biomarkers with relatively low concentrations.

Across all points in pregnancy, we observed concentrations of MEP that were 7-10% lower for women in the “low fragranced product use” group compared to the population mean. Similarly, concentrations of replacement biomarkers were lower among women in the “low fragranced product use” group compared to the population mean. Magnitude of difference ranged from 9-21% lower for ∑DiNCH and 15-21% lower for ∑DEHTP. Most other phthalate and replacement biomarker concentrations were not significantly different from the population mean among “low fragranced product use” group members; however, ∑DnBP concentrations were significantly higher among these individuals (22–38%) in early and late pregnancy.

Concentrations of other LMW biomarkers were frequently lower among women in either of the “fragranced product” groups compared to the population mean. In early pregnancy, women in both the “fragranced product and low bar soap use” and “fragranced product and low body wash use” groups had lower biomarker concentrations, with largest differences observed for ∑DiBP. In mid-pregnancy, concentrations were lower for women in the “fragranced product and low bar soap use” group but not the “fragranced product and low body wash use” group while the opposite pattern was observed for late pregnancy.

Patterns for HMW phthalate biomarkers were not as clear. However, we did note that concentrations tended to be lower among individuals from the “Fragranced product and low body wash use” group in early and late pregnancy. Estimates were imprecise, especially for MCPP and MCNP.

In our sensitivity analyses, we repeated analyses with 3 product use classes at each time point. In early and late pregnancy, the groups were subsequently labeled as, “Low fragranced product use,” “Fragranced product and low bar soap use,” and “Fragranced product and low body wash use.” In mid-pregnancy, the groups were labeled as “Low fragranced product use,” “Fragranced product use,” and “Low product use.” Associations with exposure biomarkers were not meaningfully different from the main analyses and primary findings were consistent (e.g., lower MEP, ∑DEHTP, and ∑DiNCH concentrations among women in the “Low fragranced product use” group). Rerunning the models with different subsets of products resulted in similar groups identified as in the primary model (data not shown). When we reran analyses retaining the “fragrance unknown” response, overall conclusions were similar and the associations between lower concentrations of MEP and replacement biomarkers among women in the “low fragranced product use” group remained (data not shown).

## Discussion

In this analysis of patterns of personal care product use and phthalate and replacement biomarker urinary concentrations, we observed that patterns of product use were mostly consistent across pregnancy. Women sorted into groups that generally corresponded to (1) low use of fragranced products; (2) use of fragranced products but low use of body wash; (3) use of fragranced products but low use of bar soap; (4) low use of any products, or mixed fragrance use with high cosmetics and perfume in late pregnancy. Women tended to stay in the same product use groups between early and mid-pregnancy, but less so between mid- and late pregnancy. Associations between group membership and phthalate and replacement biomarkers were generally similar within class (i.e., HMW, LMW, replacement), but findings were not altogether consistent across pregnancy. There was no one product use group that was consistently associated with lower biomarker concentrations, which is not surprising given the multitude of uses for phthalates and replacements. However, women who reported low use of fragranced products had lower concentrations of MEP and replacement biomarkers compared to the population mean. While there is not a clear recommendation for safe product use leading to uniform reduction of biomarkers concentrations, the findings between women in the “low fragranced product use” group and MEP and replacement concentrations are notable.

MEP, the main metabolite of DEP, is the phthalate biomarker most commonly associated with fragranced personal care products [30, 31]. In our population, lower concentrations of MEP were noted at all points in pregnancy among women in the “low fragranced product use” group, suggesting that minimizing use of fragranced products may indeed translate to lower exposure to DEP. This finding also serves as a proof of concept for the ability of product use groupings to replicate well-established relationships. However, lower concentrations of MEP were noted among women in the “Fragranced products and low bar soap use” in early and mid-pregnancy.

DnBP and DiBP have been used in nail polish and cosmetics [32, 33] and are the LMW phthalates most strongly and consistently associated with adverse pregnancy outcomes [6, 34, 35]. Associations between group membership and concentrations of ∑DnBP and ∑DiBP were inconsistent, although lower concentrations were observed in both “fragranced products” groups compared to the population mean at different time points.

Associations with HMW phthalates were also somewhat inconsistent. HMW phthalates are used less in personal care products and found more in food and beverage items [8], so this inconsistency is somewhat expected. Notably, some percent differences in group members compared to the population mean may be exaggerated because of relatively low baseline concentrations (e.g., MCPP, MCNP).

Interestingly, we noted consistently lower concentrations of replacement biomarkers among individuals with low fragranced use. DEHTP and DINCH are considered replacements for DEHP, a HMW phthalate. However, given the consistently lower concentrations of ∑DEHTP and ∑DINCH among women who reported low use of fragranced products, it is possible that they are also being used as replacements for other chemicals, such as LMW phthalates that are more commonly used in cosmetics and fragranced products. It must be noted that the confidence intervals for ∑DINCH are quite large, likely a result of the low baseline concentrations.

We noted some unexpected results in our study, including higher concentrations of MEP and ∑DiBP in the “Low fragranced product use” group in early pregnancy, and lower concentrations of some metabolites in the “Fragranced product” groups. Due to the lack of replication of these associations across pregnancy, we believe that these may be statistical anomalies and not representative of true trends. Given the breadth of results, we focused on those that were consistent across pregnancy.

Studying the association between use of personal care products and biomarker concentrations is important as a possible avenue for chemical exposure reduction. Personal care products, cosmetics, and fragranced items are known exposure sources for phthalates and their replacements [30]. The Food and Drug Administration does not have the same authority over cosmetics compared to food and drugs, and does not have the ability to recall products, nor do they require premarket safety testing for cosmetics, other than for color additives [36, 37]. Unlike for food, drugs, drinking water, pesticides, cars, electronics, etc., there are no U.S. governmental standards for cosmetics or personal care products [36, 37].

Information on the safety and composition of cosmetics or personal care products is largely inaccessible to the public, despite the potential for adverse effects. Because many phthalates are considered components of “fragrance” [31], they are not explicitly listed as ingredients, and thus the exact composition of many products and the presence of phthalates and their replacements are unknown. Additionally, products labeled as “fragrance-free” may still contain phthalates and their replacements for other functions and product formulations frequently change. Furthermore, there is no verification of products that specifically advertise as “phthalate-free.” Accordingly, it is difficult for consumers and researchers to know which chemicals are included in the products they use. This may be a particular problem for pregnant women, who often desire advice on how to minimize exposure to potentially dangerous chemicals [38, 39].

“Fragrance free” labeling is poorly regulated; therefore, we cannot distinguish use of fragrance-free products (not all of which may in fact be free of fragrance) from behaviors associated with purchasing products labeled “fragrance free.” We did not see strong differences between biomarker concentrations among women in this group and other product use groups, though we did observe consistently lower concentrations of MEP, a metabolite of DEP which is often found in scented products [4]. If women in this group had lower concentrations of many biomarkers, it may have reflected behavioral patterns or demographic factors associated with lower overall phthalate exposure, rather than lower concentrations driven by selected product use. Our observation of specific differences in MEP concentrations suggests the possibility that use of fragrance-free products may reduce DEP exposure, but further research is needed to confirm this finding.

Parsing out the relationship between product use and biomarker concentrations of phthalates and replacements is plagued by many issues. These include the non-persistent nature of phthalates, ubiquitous sources of phthalate exposure, lack of transparency in product content and labeling, relatively frequent formulation changes in personal care products, and use of many products simultaneously. Our choice of assessing product use classes in lieu of individual products addresses the last issue, but the other complications remain. Additionally, there are strong racial and ethnic patterns in use for some products [40, 41]. Namely, non-Hispanic Black women report significantly less use of shampoo and conditioner, which we also observed in this population. Accordingly, these items were not used to construct the product classes, as they were too collinear with race and ethnicity and subsequent product classes would be essentially racially stratified.

There is a substantial body of literature on the association between product use and phthalate biomarker concentrations, both in pregnant and non-pregnant populations, with a variety of methods used and products queried [10–19, 42–46]. Nearly all prior studies have examined one product at a time in regression models, ignoring that women frequently use multiple products simultaneously. There is little consistency in the literature regarding which products are associated with which metabolites and direction of effects. The lack of consistency in prior literature and in our study across pregnancy likely related to the analytic difficulties outlined above. The most consistent findings across studies involved MEP, which was found to be higher following use of hairspray/gel, cosmetics, “other hair products,” foundation/toner, lipstick/blush, lotion, deodorant, perfume/cologne, and sunscreen. Additional associations noted in at least two studies include lotion use and elevated MEHHP, a DEHP metabolite, eye makeup and face cream with higher MBP (main metabolite of DBP), perfume/cologne and higher MiBP (metabolite of DiBP), and hairspray/gel use with lower MCOP (metabolite of DiNP). No prior studies have examined differences in phthalate or replacement biomarker concentrations between fragranced products and fragrance-free items.

In one intervention study specifically focusing on reducing personal care product use, Harley et al. observed that using personal care products for three days that were labeled as not containing phthalates led to modest decreases in urinary MEP and MBP among Latina girls [47]. Our findings suggest that an intervention focused on avoiding fragranced products could be an alternative to reducing exposure to DEP. However, this group of individuals in our study also had higher ∑DnBP concentrations in early and late pregnancy, which is not desirable.

The groups identified in our study reflect real and meaningful differences in product use patterns. Two of the groups were quite similar and differed only with regard to whether women tended to use bar soap or body wash. In mid-pregnancy, women in the group corresponding to low body wash use had significantly higher concentrations of MEP than women in the low bar soap group, however, this association was not repeated at other points in pregnancy. Other associations with biomarker concentrations tended to be similar across these two groups, suggesting that the overall fragranced use of products was a stronger predictor of biomarker concentrations and potentially a better target for intervention than the specific combination of fragranced products.

Previous literature has largely not examined changes in product usage over pregnancy. We were interested in changes in group membership to understand how pregnancy progression might influence product use and subsequently exposure to phthalates and replacements. Changes in group membership over time may be explained by concerns about safety, scent aversions, variable skin sensitivities, or differences in purchasing habits, among others. However, our ability to assess the link between group changes and corresponding changes in biomarker concentrations was hampered by the few consistent associations observed between product use groups and biomarker concentrations, as well as the considerable consistency in group membership between early and mid-pregnancy. This may be due to the shorter time elapsed between visits (i.e., visits in early pregnancy were closer in time to those in mid-pregnancy than visits in mid-pregnancy and late pregnancy). There were more changes in group membership between mid- and late pregnancy but this is likely related to the differences in group profiles in late pregnancy. We did not identify any notable demographic differences in women who remained in the same product use group between consecutive time points vs. those whose use patterns resulted in different group memberships.

There are some limitations to this study. To improve the accuracy of biomarker measures, we created averaged measures of repeated phthalate or alternative metabolite measurements within pregnancy periods. However, doing so also required the creation of composite questionnaire responses within windows. Because we could not easily average dichotomous variables in this step, we opted to use the first available response. This may have resulted in some exposure misclassification, but the use of a pooled value for biomarkers helps mitigate this concern as it reflects biomarker concentrations over the entire window [25, 48]. Additionally, concordance in reported product use was relatively high within pregnancy window. Furthermore, when biomarker concentrations are relatively low, percent difference may overstate findings; examination of confidence intervals thus helps to contextualize results. Lastly, though our use of product groupings address confounding within personal care products, it does not account for confounding across product sources. For example, diet is a meaningful source of phthalate exposure [8] and may also be associated with covariates that affect product use. Though we attempted to address this by weighting our estimates of biomarker concentrations with demographic variables, residual confounding is possible.

However, this study also contained many strengths. Our use of LCA to group women is likely a better representation of the true way that women experience exposures, as women rarely use exclusively one product and examining them individually ignores the simultaneous use. Additionally, most previous studies simply ask about use of a product (yes/no) without consideration of its fragrance status. Given that phthalates are frequently used as scent stabilizers, the fragranced status of an item is likely an important determinant of phthalate exposure. Prior studies also largely did not have available information on product formulation or brand. While this information was also missing from this present analysis, the use of groupings helped circumvent this concern, as fragrance-free versions of a product will likely be more similar in formulation than a fragrance-free item compared to a fragranced item. Lastly, using averages for biomarker concentrations within pregnancy period improves the accuracy of the measure compared to a single spot measurement when trying to capture an extended pregnancy window (i.e., trimester).

In this study using a racially and socioeconomically diverse cohort of pregnant women, we did not find strong evidence to support adhering to a certain pattern of personal care product use to avoid phthalate or replacement exposure, despite applying an innovative approach to address simultaneous product use, as well as robust exposure biomarker data. However, we did note that MEP and replacement biomarkers concentrations were consistently lower among women in the “low fragranced product use” group. Though personal care products and cosmetics are a known source of phthalate exposure, epidemiological studies linking the two have only consistently identified associations with MEP. Phthalate exposure sources are diverse and ubiquitous and thus trying to narrow down exposure from personal care products and determine associations is challenging. Given these difficulties, exploring alternative options for reduction of phthalate exposures, potentially through policy measures or voluntary action by manufacturers, may be merited.

## Disclaimer

The findings and conclusions in this report are those of the authors and do not necessarily represent the official position of the Centers for Disease Control and Prevention. Use of trade names is for identification only and does not imply endorsement by the CDC, the Public Health Service, or the US Department of Health and Human Services.

### Supplementary information

Original Research Article

## Data Availability

Phthalate exposure biomarker data is available from the corresponding author, KKF, upon reasonable request.
