# Supplementary material for: Personal care product use patterns in association with phthalate and replacement biomarkers across pregnancy
Source: J Expo Sci Environ Epidemiol. 2024 Jan 4;34(4):591–600. doi: 10.1038/s41370-023-00627-w (PMC11303244; doi:10.1038/s41370-023-00627-w)

Original Research Article

Title: Personal care product use patterns in association with phthalates and replacements over gestation

Authors: Emma M. Rosen[a,b], Danielle R. Stevens[a], Amanda M. Ramos[b], Erin E. McNell[a], Mollie E. Wood[b], Stephanie M. Engel[b], Alexander P. Keil[c], Antonia M Calafat[d], Julianne Cook Botelho[d], Elena Sinkovskaya[e], Ann Przybylska[e], George Saade[f], Alfred Abuhamad[e], Kelly K. Ferguson[a]

[a]Epidemiology Branch, National Institute of Environmental Health Sciences, Durham, North Carolina, USA

[b]Department of Epidemiology, University of North Carolina-Chapel Hill, Chapel Hill, North Carolina, USA

[c]Division of Cancer Epidemiology and Genetics, National Cancer Institute, Bethesda, MD, USA

[d]Division of Laboratory Sciences, National Center for Environmental Health, Centers for Disease Control and Prevention, Atlanta, Georgia, USA

[e]Department of Obstetrics and Gynecology, Division of Maternal-Fetal Medicine, Eastern Virginia Medical School, Norfolk, Virginia, USA

[f]Department of Obstetrics and Gynecology, University of Texas Medical Branch, Galveston, Texas, USA

Table of Contents

Supplemental table 1. Personal care products included in questionnaire 3

Supplemental table 2. Detection frequency of phthalate and replacement metabolites in all urine samples analyzed (n=1866) in the Human Placenta and Phthalates Study (2017-2018)..................5

Supplemental table 3. Percent detection of measured metabolites at each visit...............................6

Supplemental table 4. Fit statistics for LCA groups 7

Supplemental table 5. Transition probabilities between latent class groups from early pregnancy to mid-pregnancy...8

Supplemental table 6. Transition probabilities between latent class groups from mid-pregnancy to late pregnancy 8

Supplemental table 7. Transition probabilities between latent class groups from early pregnancy to late pregnancy 9

Supplemental table 8. Demographic comparison of women who were classified into different product use groups across pregnancy periods, N (%) 10

Supplemental figure 1. Decision tree for product use classification..11

Supplemental figure 2. Sankey plot demonstrating product use transitions across pregnancy 12

Supplemental table 1. Personal care products included in questionnaire

Product	Initial screening	Treated for analysis
Deodorant		Frag / FF / no use
Bar soap (solid)		Frag / FF / no use
Body wash (liquid)		Frag / FF / no use
Liquid or foaming hand soap	combined into one variable	Frag / FF / no use
Liquid soaps		
Lotion		Frag / FF / no use
Shaving cream		Included in sensitivity analysis
Sunscreen		Included in sensitivity analysis
Facial cleanser		Frag / FF / no use
Fingernail polish		Included in sensitivity analysis
Colored cosmetics (e.g., foundation, blush, eye makeup)	combined into one variable	Any use / no use
Lip cosmetics (e.g., lipstick, lipgloss, chapstick, lip balm, Vaseline)		
Shampoo		Dropped due to racial collinearity
Conditioner	combined into one variable	Dropped due to racial collinearity
Leave-in conditioner		
Hair crème		Excluded due to low reported use
Hair spray/hair gel		Frag / FF / no use
Anti-frizz or polishing products		Excluded due to low reported use
Root stimulators	Dropped due to <10 women reporting use	
Hair lotion	Dropped due to <10 women reporting use	
Hair relaxer or straightener	Dropped due to <10 women reporting use	
Hair perm	Dropped due to <10 women reporting use	
Hair bleaches or dyes	Dropped due to <10 women reporting use	
Hair oil		Excluded due to low reported use
Other hair products		Excluded due to low reported use
Perfume		Any use / no use
Mouthwash		Included in sensitivity analysis
Feminine spray	Dropped due to <10 women reporting use	
Feminine powder		Excluded due to low reported use
Feminine wipes/towelettes		Excluded due to low reported use
Vaginal douches	Dropped due to <10 women reporting use	
Lubricants	Dropped due to <10 women reporting use	
Anti-itch creams or yeast infection treatments	Dropped due to <10 women reporting use	

Abbreviations: fragrance free (FF)

Supplemental table 2. Detection frequency of phthalate and replacement metabolites in all urine samples analyzed (n=1866) in the Human Placenta and Phthalates Study (2017-2018)

**Phthalate metabolites**	Biomarker abbreviation	LOD	% Detection
Monoethyl phthalate	MEP	1.2	99.6
Sum of di-n-butyl phthalate	*∑DnBP*		
Mono-n-butyl phthalate	MBP	0.4	96.8
Mono-hydroxybutyl phthalate	MHBP	0.4	76.7
Sum of di-iso-butyl phthalate	*∑DiBP*		
Mono-hydroxybutyl phthalate	MiBP	0.8	98.9
Mono-hydroxy-isobutyl phthalate	MHiBP	0.4	98.5
Monobenzyl phthalate	MBzP	0.3	98.0
Mono-3-carboxypropyl phthalates	MCPP	0.4	73.2
Sum of di(2-ethylhexyl) phthalate	*∑DEHP*		
Mono-2-ethylhexyl phthalate	MEHP	0.8	61.5
Mono-2-ethyl-5-hydroxyhexyl phthalate	MEHHP	0.4	99.1
Mono-2-ethyl-5-oxohexyl phthalate	MEOHP	0.2	99.8
Mono-2-ethyl-5-carboxypentyl phthalate	MECPP	0.4	99.9
Sum of di-isononyl phthalate	*∑DiNP*		
Mono oxononyl phthalate	MONP	0.4	91.0
Mono carboxyisooctyl phthalate	MCOP	0.3	99.7
Mono carboxyisononyl phthalate	MCNP	0.2	95.2
**Replacement metabolites**			
Sum of di(2-ethylhexyl) terephthalate	*∑DEHTP*		
Mono-2-ethyl-5-hydrohexyl terephthalate	MEHHTP	0.4	98.9
Mono-2-ethyl-5-carboxypentyl terephthalate	MECPTP	0.2	100
Sum of 1,2-cyclohexane dicarboxylic acid, diisononyl ester	*∑DiNCH*		
Cyclohexane-1,2-dicarboxylic acid, monohydroxy isononyl ester	MHiNCH	0.4	74.8
Cyclohexane-1,2-dicarboxylic acid, monocarboxy isooctyl ester	MCOCH	0.5	42.7

Abbreviations: Limit of detection (LOD)

Supplemental table 3. Percent detection of measured metabolites at each visit

Metabolite	LOD (ng/mL)	Visit 1 (n=237)	Visit 2 (n=235)	Visit 3 (n=239)	Visit 4 (n=231)	Visit 5 (n=235)	Visit 6 (n=235)	Visit 7 (n=247)	Visit 8 (n=207)
MEP	1.2	100	98.7	99.6	100	99.2	99.6	100	100
MBP	0.4	95.8	95.7	97.5	97	94.5	97	99.2	98.1
MHBP	0.4	70.9	76.2	77	76.6	77.5	77.9	80.6	76.6
MiBP	0.8	98.7	99.2	97.9	100	97.5	99.2	99.6	99.5
MHiBP	0.4	97.1	97.9	97.1	99.1	97.9	100	99.6	99
MBzP	0.3	98.3	97.9	97.9	97.8	96.2	99.2	98.4	98.1
MCPP	0.4	81.4	86.8	71.1	71.4	69.4	78.3	60.3	67
MEHP	0.8	58.2	68.9	64	63.2	66.2	60	55.1	56.5
MEHHP	0.4	99.6	99.2	98.7	98.7	98.7	99.2	99.6	99.5
MEOHP	0.2	99.6	100	99.6	100	99.6	100	100	100
MECPP	0.4	100	99.6	99.6	100	100	100	100	100
MONP	0.4	86.5	92.3	90.8	90.9	87.2	94.9	90.7	94.7
MCOP	0.3	100	100	99.2	98.7	100	100	100	100
MCNP	0.2	94.1	96.2	96.2	95.7	93.2	96.6	93.1	96.7
MEHHTP	0.4	99.6	100	99.2	99.1	98.7	98.7	98	98.1
MECPTP	0.2	100	100	100	100	100	100	100	100
MHiNCH	0.4	76.8	76.6	77.8	79.2	73.6	80	66.4	67.5
MCOCH	0.5	40.5	47.7	51.5	48.1	34.9	42.6	35.2	41.2

Abbreviations: limit of detection (LOD); monoethyl phthalate (MEP); mono-n-butyl phthalate (MBP); mono-hydroxybutyl phthalate (MHBP), mono-isobutyl phthalate (MiBP); mono-hydroxy-isobutyl phthalate (MHiBP); monobenzyl phthalate (MBzP); mono-3-carboxypropyl phthalate (MCPP); mono-2-ethylhexyl phthalate (MEHP); mono-2-ethyl-5-hydroxyhexyl phthalate (MEHHP); mono-2-ethyl-5-oxohexyl phthalate (MEOHP); mono-2-ethyl-5-carboxypentyl phthalate (MECPP); mono oxononyl phthalate (MONP); mono carboxyisooctyl phthalate (MCOP); mono carboxyisononyl phthalate (MCNP); mono-2-ethyl-5-hydrohexyl terephthalate (MEHHTP); mono-2-ethyl-5-carboxypentyl terephthalate (MECPTP); cyclohexane-1,2-dicarboxylic acid, monohydroxy isononyl ester (MHiNCH); cyclohexane-1,2-dicarboxylic acid, monocarboxy isooctyl ester (MCOCH)

Supplemental table 4. Fit statistics for LCA groups

Number of groups	Log likelihood	G-squared	AIC	ABIC	Entropy	Median posterior probabilities	Group membership percentages
Early pregnancy							
2	-1612.89	885.11	951.11	963.73	0.685	0.973 / 0.987	32.6 / 67.4
3	-1559.1	777.54	877.54	896.67	0.763	0.972 / 0.997 / 0.962	20.5 / 49.6 / 29.8
**4**	**-1532.59**	**724.52**	**858.52**	884.16	0.789	**0.987 / 0.910 / 0.939 / 0.970**	**16.3 / 51.2 / 25.6 / 7.0**
5	-1511.8	682.93	850.93	883.35	0.754	0.960 / 0.985 / 0.864 / 0.952 / 0.960	9.3 / 16.3 / 12.4 / 23.3 / 38.8
Mid-pregnancy							
2	-1726.77	930.23	996.23	1010.10	0.760	0.989 / 0.997	22.0 / 78.0
3	-1680.59	837.88	937.88	958.90	0.703	0.890 / 0.932 / 0.997	38.4 / 41.4 / 20.1
**4**	**-1642.02**	**760.75**	**894.75**	**922.91**	**0.777**	**0.962 / 0.999 / 0.940 / 0.943**	**23.5 / 16.8 / 33.6 / 26.1**
5	-1617.06	710.81	878.81	915.16	0.793	0.999 / 0.982 / 0.978 / 0.938 / 0.931	10.1 / 20.5 / 9.7 / 33.6 / 26.1
Late pregnancy							
2	-1708.11	887.18	953.18	967.66	0.824	0.994 / 0.999	26.0 / 74.0
3	-1659.14	789.26	889.26	911.19	0.787	0.990 / 0.964 / 0.995	50.9 / 24.2 / 24.9
**4**	**-1621.37**	**713.72**	**847.72**	**877.11**	**0.828**	**0.994 / 0.983 / 0.948 / 0.977**	**23.1 / 45.1 / 22.0 / 9.9**
5	-1600.91	672.80	840.8	877.65	0.790	0.996 / 0.835 / 0.921 / 0.979 / 0.978	23.1 / 27.8 / 17.2 / 9.9 / 22.0

Abbreviations: latent class analysis (LCA); Akaike information criterion (AIC); Adjusted Bayesian information criterion (ABIC)

Note: bolded rows indicate selected models

Supplemental table 5. Transition probabilities between latent class groups from early pregnancy to mid-pregnancy

	Fragranced product and low bar soap use	Fragranced product and low body wash use	Low fragranced product use	Low product use
Fragranced product and low bar soap use	0.77	0.11	0.11	0
Fragranced product and low body wash use	0	0.92	0.08	0
Low fragranced product use	0	0.07	0.62	0.31
Low product use	0.24	0	0	0.76

Probabilities displayed are row percentages describing the proportion of women from each assigned class in early pregnancy (row) who subsequently transition to each assigned class in mid-pregnancy (column).

Supplemental table 6. Transition probabilities between latent class groups from mid-pregnancy to late pregnancy

	Fragranced product and low bar soap use	Fragranced product and low body wash use	Low fragranced product use	Mixed use; high cosmetics/ perfume
Fragranced product and low bar soap use	0.84	0.12	0.04	0
Fragranced product and low body wash use	0	0.24	0.09	0.67
Low fragranced product use	0.25	0.09	0.51	0.15
Low product use	0	0.48	0.52	0

Probabilities displayed are row percentages describing the proportion of women from each assigned class in mid-pregnancy (row) who subsequently transition to each assigned class in late pregnancy (column)

Supplemental table 7. Transition probabilities between latent class groups from early pregnancy to late pregnancy

	Fragranced product and low bar soap use	Fragranced product and low body wash use	Low fragranced product use	Mixed use; high cosmetics/ perfume
Fragranced product and low bar soap use	0.65	0.16	0.14	0.06
Fragranced product and low body wash use	0	0.18	0	0.82
Low fragranced product use	0.31	0	0.55	0.14
Low product use	0.07	0.30	0.64	0

Probabilities displayed are row percentages describing the proportion of women from each assigned class in early pregnancy (row) who subsequently transition to each assigned class in late pregnancy (column)

Supplemental table 8. Demographic comparison of women who were classified into different product use groups across pregnancy periods, N (%)

		Women who were classified into different product use groups	
	Overall population (n=303)	Between early and mid-pregnancy (n=44)	Between mid- and late pregnancy (n=158)
**Race/ethnicity**			
Non-Hispanic White	118 (38.9)	12 (27.3)	47 (29.8)
Non-Hispanic Black	131 (43.2)	24 (54.5)	81 (51.3)
Hispanic	49 (16.2)	8 (18.2)	27 (17.1)
Other[1]	5 (1.7)	0 (0)	3 (1.9)
**Clinic Site**			
EVMS	218 (72.0)	31 (70.5)	112 (70.9)
UTMB	85 (28.1)	13 (29.5)	46 (29.1)
**Marital status**			
Single[2]	131 (43.2)	21 (50.0)	78 (50.3)
Married/living with partner	166 (54.8)	21 (50.0)	77 (49.7)
Missing	6	2	3
**Current employment**			
None	139 (48.1)	20 (47.6)	73 (48.0)
Any	150 (51.9)	22 (52.4)	79 (52.0)
Missing	14	2	6
**Health insurance[3]**			
Private	82 (27.5)	12 (27.9)	34 (21.8)
Govnt-assisted	216 (72.5)	31 (72.1)	122 (78.2)
Missing	5	1	2
**Parity**			
0	108 (35.8)	14 (31.8)	53 (33.8)
1-2	155 (51.3)	26 (59.1)	81 (51.6)
3+	39 (12.9)	4 (9.1)	23 (14.7)
**Smoking in 3 months prior to pregnancy**			
No	204 (67.8)	28 (63.6)	109 (69.4)
Yes	97 (32.2)	16 (36.4)	48 (30.6)
Missing	2	0	1
**Education**			
High school or below	131 (44.7)	17 (38.6)	74 (47.1)
Some college, technical school, or associates	123 (42.0)	25 (56.8)	67 (42.7)
4-year degree	39 (13.3)	2 (4.6)	16 (10.2)
Missing	10	0	1
**Age** (years)			
18-22	78 (25.9)	11 (26.2)	45 (28.9)
23-26.5	83 (27.6)	8 (19.1)	38 (24.4)
27-30	72 (23.9)	14 (33.3)	42 (26.9)
30-46	68 (22.6)	9 (21.4)	31 (19.9)
Missing	2	2	2
**Early pregnancy BMI**			
<18.5	16 (5.3)	3 (6.8)	9 (5.7)
18.5-24.99	135 (44.7)	23 (52.3)	71 (44.9)
25-29.99	112 (37.1)	15 (34.1)	59 (37.3)
>30	39 (12.9)	3 (6.8)	19 (12.0)
Missing	1	0	0

Supplemental figure 1. Decision tree for product use classification

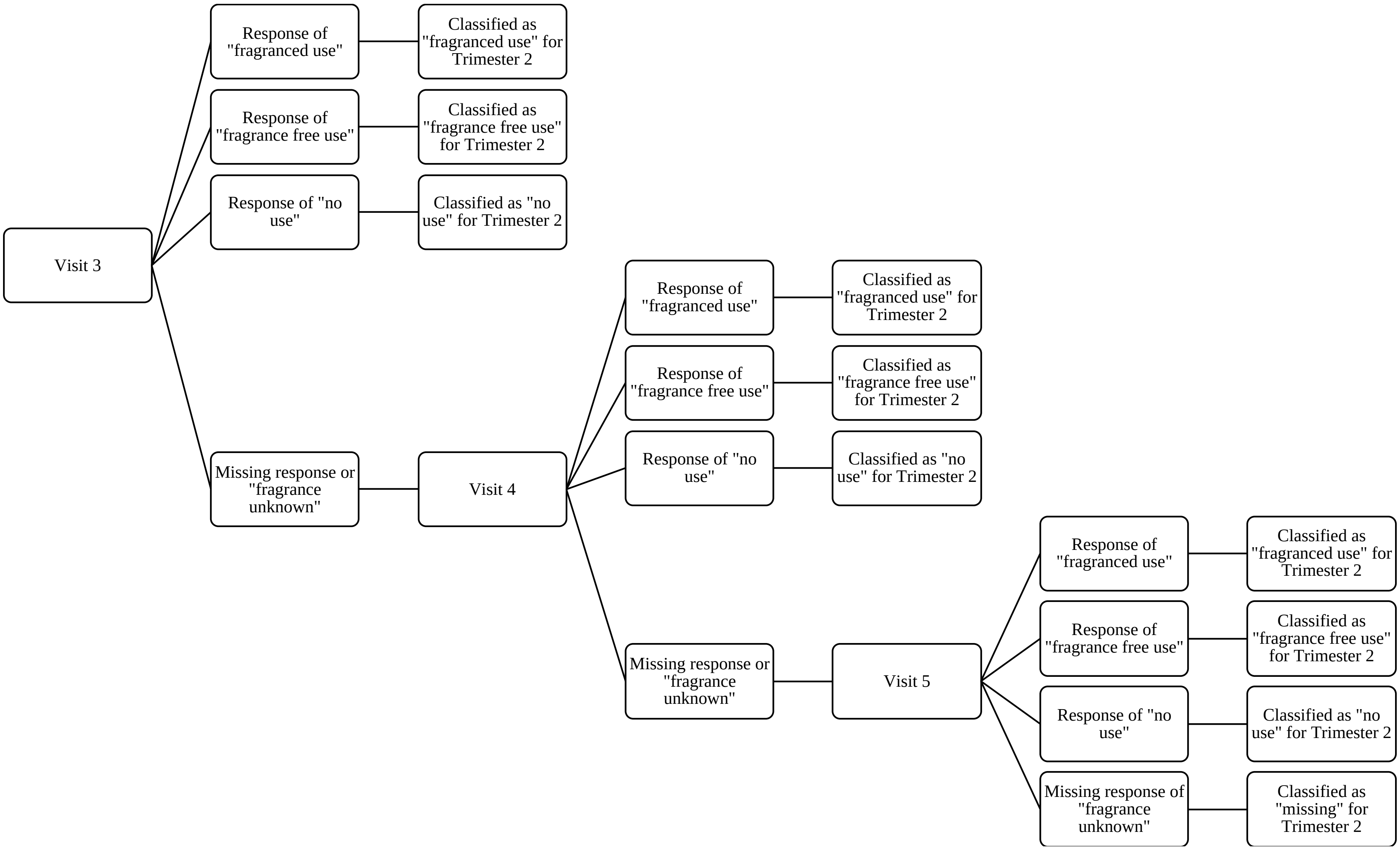

Supplemental figure 2. Sankey plot demonstrating product use transitions across pregnancy

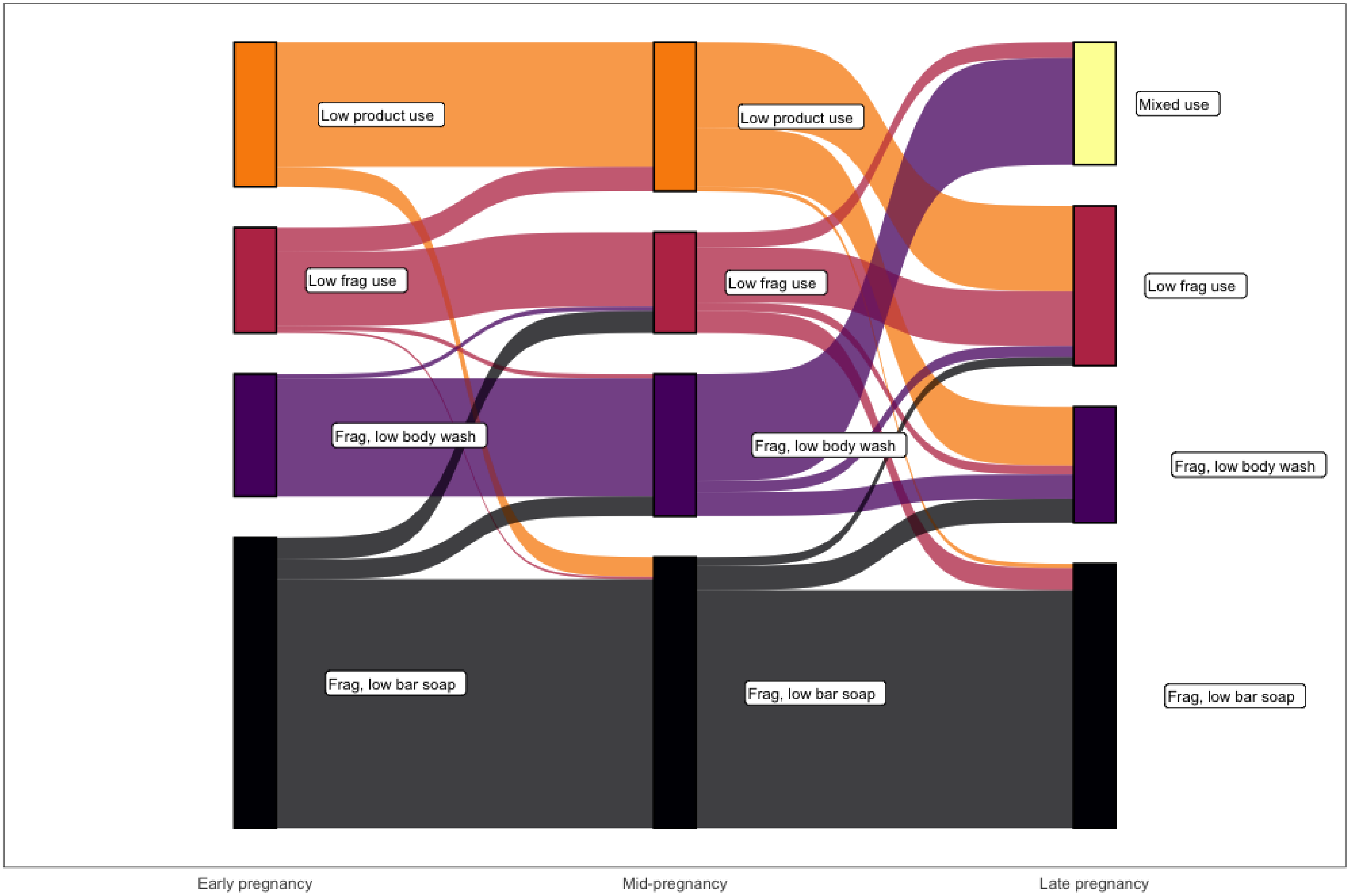

Supplement: Supplementary file 1 — Original Research Article [file 41370_2023_627_MOESM1_ESM.pdf]
